# Voltage-Dependent Inhibition of Glycine Receptor Channels by Niflumic Acid

**DOI:** 10.3389/fnmol.2017.00125

**Published:** 2017-05-16

**Authors:** Galyna Maleeva, Franck Peiretti, Boris S. Zhorov, Piotr Bregestovski

**Affiliations:** ^1^INSERM, INS, Institut de Neurosciences des Systèmes, Aix-Marseille UniversityMarseille, France; ^2^Department of Cytology, Bogomoletz Institute of PhysiologyKyiv, Ukraine; ^3^INSERM 1062, INRA 1260, NORT, Aix-Marseille UniversityMarseille, France; ^4^Sechenov Institute of Evolutionary Physiology and Biochemistry, Russian Academy of SciencesSt. Petersburg, Russia; ^5^Department of Biochemistry and Biomedical Sciences, McMaster UniversityHamilton, ON, Canada; ^6^Department of Physiology, Kazan State Medical UniversityKazan, Russia

**Keywords:** chloride-permeable channels, patch-clamp recordings, cys-loop receptors, Woodhull analysis, Monte Carlo energy minimizations

## Abstract

Niflumic acid (NFA) is a member of the fenamate class of nonsteroidal anti-inflammatory drugs. This compound and its derivatives are used worldwide clinically for the relief of chronic and acute pain. NFA is also a commonly used blocker of voltage-gated chloride channels. Here we present evidence that NFA is an efficient blocker of chloride-permeable glycine receptors (GlyRs) with subunit heterogeneity of action. Using the whole-cell configuration of patch-clamp recordings and molecular modeling, we analyzed the action of NFA on homomeric α1ΔIns, α2B, α3L, and heteromeric α1β and α2β GlyRs expressed in CHO cells. NFA inhibited glycine-induced currents in a voltage-dependent manner and its blocking potency in α2 and α3 GlyRs was higher than that in α1 GlyR. The Woodhull analysis suggests that NFA blocks α1 and α2 GlyRs at the fractional electrical distances of 0.16 and 0.65 from the external membrane surface, respectively. Thus, NFA binding site in α1 GlyR is closer to the external part of the membrane, while in α2 GlyR it is significantly deeper in the pore. Mutation G254A at the cytoplasmic part of the α1 GlyR pore-lining TM2 helix (level 2′) increased the NFA blocking potency, while incorporation of the β subunit did not have a significant effect. The Hill plot analysis suggests that α1 and α2 GlyRs are preferably blocked by two and one NFA molecules, respectively. Molecular modeling using Monte Carlo energy minimizations provides the structural rationale for the experimental data and proposes more than one interaction site along the pore where NFA can suppress the ion permeation.

## Introduction

The main inhibitory drive in mammalian CNS is provided by chloride (Cl^−^)-permeable GABA_A_- and glycine receptors (GlyRs) (Sigel and Steinmann, 2012; Lynagh and Pless, 2014). These transmembrane proteins belong to the superfamily of pentameric Cys-loop ligand-gated channels, which also includes cation-selective nicotinic acetylcholine receptor and serotonin type3 receptor (Betz, 1990; Miller and Smart, 2010). GlyRs are predominantly expressed in the spinal cord (Young and Snyder, 1973), in the brain stem (Frostholm and Rotter, 1985; Probst et al., 1986), cerebellum (Garcia-Alcocer et al., 2008), other higher brain regions (Bristow et al., 1986), and in retina (Haverkamp et al., 2003). Functionally GlyRs participate in the movement control, perception of visual, acoustic and sensory signals and pain sensation (Harvey et al., 2004; Betz and Laube, 2006). Dysfunction of these receptors is associated with hyperekplexia and temporal lobe seizures accompanied by memory deficits (Lynch, 2009; Schaefer et al., 2012; Zuliani et al., 2014). In the nervous system of vertebrates, molecular cloning identified four genes encoding alpha (α1–α4) subunits and one single gene encoding beta GlyR subunits (Grenningloh et al., 1987, 1990; rev. Dutertre et al., 2012). These subunits assemble to form homopentameric α GlyRs and heteropentameric α/β GlyRs (Lynch, 2004).

**Figure 1:**
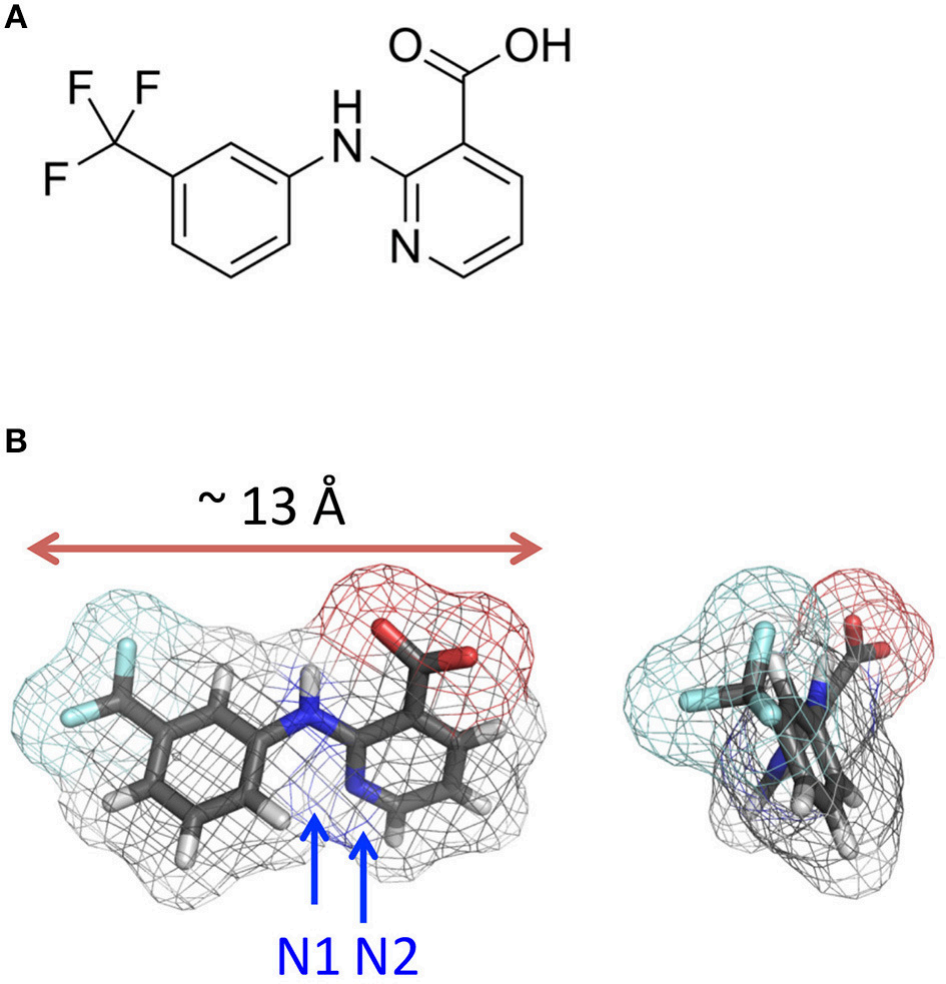
**Niflumic acid. (A)** Structural formula. **(B)** Orthogonal views of the 3D structure. The distance between most remote atoms is 10.6 Å and the maximal distance between van der Waals surfaces of these atoms (the maximal profile) is ~ 13 Å.

Niflumic acid (NFA) (Figure 1) is a member of the fenamate class of nonsteroidal anti-inflammatory drugs originally developed for the treatment of rheumatic disorders. This drug and its derivatives are used worldwide clinically for the relief of chronic and acute pain conditions (Vincent et al., 1999; Kang et al., 2008; Cremonesi and Cavalieri, 2015). As a compound with anti-inflammatory, antipyretic, and analgesic therapeutic activity, NFA has been successfully used in clinical trials in adults (Sauvage et al., 1990; Mero et al., 2013) and children (Manach and Ditisheim, 1990; Lantz et al., 1994; Sturkenboom et al., 2005). The primary mechanism of NFA action is the inhibition of enzymes involved in the synthesis of proinflammatory prostaglandins (Smith, 1992; McCarberg and Gibofsky, 2012): cyclooxygenase (prostaglandin synthase) (Barnett et al., 1994; Johnson et al., 1995) and phospholipase A2 (PLA_2_) (Jabeen et al., 2005). The structure of the complex of PLA_2_ with NFA has been determined at the 2.5 Å resolution revealing residues in the substrate-binding hydrophobic channel of the enzyme (Jabeen et al., 2005).

NFA is also known as a modulator, mainly inhibitor, of different types of anion-permeable channels. However, mechanism of its action on these proteins remains unclear. NFA blocks voltage-gated chloride channels, CLC-1 (Liantonio et al., 2007), as well as Ca^2+^-activated Cl^−^- channels (CaCCs) (White and Aylwin, 1990; Yang et al., 2008; Huanosta-Gutierrez et al., 2014). The effect of NFA on CaCCs is voltage-independent and it blocks the channels when applied from either outside or inside the cell (Qu and Hartzell, 2001). A recent study demonstrated the voltage-independence of NFA action on TMEM16A-encoded CaCCs but did not determine the site of its action, suggesting the pore-blocking or/and allosteric mechanisms (Ni et al., 2014).

A synthetic peptide corresponding to the TM2 transmembrane helix of GlyR forms functional ion channels in lipid bilayer, which can be blocked by NFA (Reddy et al., 1993). However, electrophysiological analysis on cells and subunit specificity of this interaction were not performed.

The aim of this study was to clarify the molecular mechanism of NFA action on GlyRs. We expressed GlyRs in different subunit combinations in CHO cells and recorded glycine-induced ionic currents under NFA application using the patch-clamp technique. The NFA action at different concentrations and membrane potentials in homomeric GlyRs (formed by α1–α3 subunits) and heteromeric GlyRs (formed by α1β or α2β subunits) were analyzed. We found that the apparent NFA affinity, the voltage-dependence and the depth of its binding in the membrane strongly depend on the GlyR subunit composition, which possess different TM2 transmembrane helices.

We further used molecular modeling with Monte Carlo energy minimizations to compute energy profile of NFA in the pore of α1 and α2 GlyRs. The calculations suggest that NFA can bind at more than one site within the pore. Based on our experimental data, suggesting that two and one NFA molecules block ion permeation through α1 GlyR and α2 GlyR channels, respectively, we elaborated models of NFA-bound α1 and α2 GlyRs.

## Materials and methods

### Cell culture and transfection

The experiments were carried out on cultured Chinese hamster ovary (CHO) cells obtained from the American Type Tissue Culture Collection (ATCC, Molsheim, France) that were maintained in culture conditions as previously described (Mukhtarov et al., 2013; Maleeva et al., 2015).

For electrophysiological analysis cells were transfected with cDNAs of different subunits of glycine receptor (α1ΔIns, α2B, α3L, α1-G254A, and β). One day before transfection, cells were plated on the cover slips (12 mm in diameter) and placed inside 35-mm cell culture dishes with 2 ml of medium. Transfection was performed using the Lipofectamine 3000 protocol (Life Technology, USA). To facilitate identification of transfected cells a green fluorescent protein (GFP) was added to the transfection mixture. For expression of functional heteromeric receptors, cells were simultaneously transfected with cDNAs of α and β subunits in the ratio 1:10. Three hours after the initial exposure of cells to the cDNAs the culture medium was replaced with that containing strychnine (1 μM), which prevents spontaneous activation of GlyRs. Electrophysiological recordings were performed in the fluorescent cells 24–72 h after transfection.

### Electrophysiological recordings

**Figure 2:**
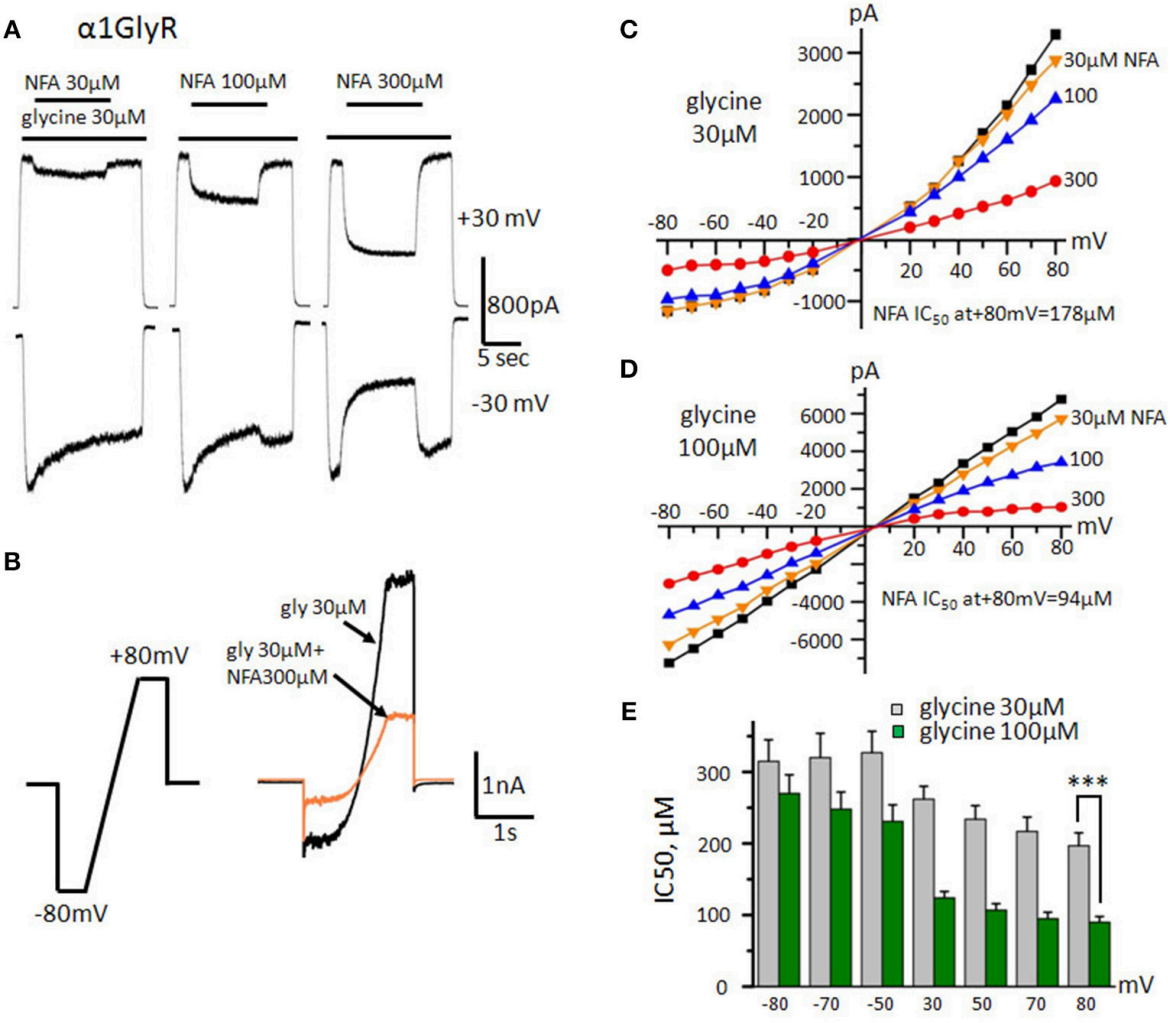
**Effect of NFA on homomeric GlyRs formed by α1 subunits. (A)** Inhibition of glycine-evoked currents (30 μM) by different concentrations of NFA (30, 100, 300 μM). Glycine was applied for 2 and 5 s at the beginning and the end of the trace respectively. In the middle of the trace, the mixture of glycine with NFA was applied (10 s). Durations of drugs applications are indicated by bars above the traces. Recordings were performed at V_hold_ +30 mV (upper traces) and −30 mV (bottom traces). **(B)** Scheme of the “ramp protocol” and representative traces obtained using this protocol in control (30 μM glycine, black) and while applying a mixture of glycine 30 μM and NFA 300 μM (orange). **(C)** Representative current-voltage relationships obtained during application of 30 μM of glycine alone (black) or mixed with different concentrations of NFA (30 μM-yellow, 100-blue, 300-red). **(D)** Representative current-voltage relationships recorded in the presence of 100 μM of glycine alone or mixed with different concentrations of NFA (30, 100, 300 μM). **(E)** NFA IC_50_ at different holding potentials. Currents were evoked by application of 30 μM (gray columns) and 100 μM (green columns, *n* = 10) of glycine. Recordings were performed using the “ramp protocol,” data represented as mean ± SEM, ^***^p < 0.001, unpaired *t*-test.

Whole-cell and outside-out recordings were performed at room temperature (20–25°C) using an EPC-9 amplifier (HEKA Elektronik, Germany). Cells were continuously superfused with external solution containing (mM): NaCl 140, CaCl_2_ 2, KCl 2.8, MgCl_2_ 4, HEPES 20, glucose 10; pH 7.4; 320–330 mOsm. Intracellular solution used for filling recording patch pipettes contained (mM): CsCl 140, CaCl_2_ 6, MgCl_2_ 2, MgATP 2, NaGTP 0.4, HEPES/CsOH 10, BAPTA (tetrapotassium salt) 2; pH 7.3; 290 mOsm. Two different protocols of solutions application were used in this study. A “long protocol” was designed to obtain recordings of current induced by subsequent application of glycine (2 s)/glycine+NFA (10 s)/glycine (5 s) on the same trace; holding potential (V_hold_) was fixed at −30 or +30 mV. A “ramp protocol” implies the gradual change of V_hold_ alternately from −80 to +80 mV and from +80 to −80 mV during 1 s, V_hold_ was fixed at −80 or +80 mV at the beginning and at the end of the ramp for 500 ms (Figure 2B).

Nonsaturating and subsaturating concentrations of glycine for different subunit combinations of GlyRs were chosen according to the EC_50_ curves obtained in previous studies. It was shown that for homomeric α1 GlyRs EC_50_ varies between 25 and 40 μM (Zhang et al., 2008; Lynagh et al., 2011; Maleeva et al., 2015), thus nonsaturating concentration of the agonist used for α1GlyRs was 30 μM, subsaturating – 100 μM. For α2 GlyRs mean value of EC_50_ for glycine was estimated as 42 ± 2 μM (Maleeva et al., 2015), i.e., close to that for α1 GlyRs, so subsaturating concentrations of glycine were the same as for α1 GlyRs. The sensitivity of α3 GlyRs to glycine is significantly lower than of α1 and α2 GlyRs, comprising 80–150 μM (Zhang et al., 2008; Maleeva et al., 2015; Sánchez et al., 2015); according to this we used 100 μM of glycine as nonsaturating concentration of the agonist. Sensitivity of α1β GlyRs is close to that of homomeric α1 GlyRs, i.e., between 25 and 50 μM (Rundstrom et al., 1994; Shan et al., 2001; Islam and Lynch, 2012); EC_50_ of α2β GlyRs varies between 50 and 85 μM (Pribilla et al., 1992; Miller et al., 2005; Zhang et al., 2008). It was demonstrated previously (Shan et al., 2001; Islam and Lynch, 2012) and confirmed by our experiments that mutation G254A does not change sensitivity of α1 GlyRs to glycine.

Recording pipettes were pulled from borosilicate glass capillaries (Harvard Apparatus Ltd, USA) and had resistances of 5–10 MOhm. For the rapid replacement of the solutions, the fast application system was used. Three parallel rectangular tubes (100 × 100 μm) were positioned 40–50 μm above the recorded cell. The movement of the tubes was controlled by a computer-driven fast exchange system (SF 77A Perfusion Fast-Step, Warner, USA) allowing a 10–90% solution exchange in 3–5 ms, as measured by open electrode controls (1/10 external solution/water). Cells with low input resistance (<150 MOhm) and a rapid run-down (>30% with repetitive application) were excluded from analysis.

### Drugs

All the drugs were obtained from Tocris or Sigma–Aldrich (France). NFA (100 mM) and picrotoxin (50 mM) were first dissolved in DMSO and then diluted with the extracellular solution to the final concentrations. In the test experiments, DMSO itself had no effect on the glycine-induced current (data not shown; see also Mascia et al., 1996; Hall et al., 2004). A stock solution of glycine (1M) was prepared using MilliQ water.

### Data analysis and statistics

Electrophysiological recordings were performed using PatchMaster (HEKA Electronic, Germany) software. To plot concentration-response curves, responses to different concentrations of glycine and NFA were fitted using a nonlinear fitting routine of the Origin 7.5 software (OriginLabs, USA) with the Hill equation:

$$\begin{array}{l} \text{For glycine}:I=Imax/{\left(1+({\text{EC}}_{50}/[\text{A}]\right)}^{nH})\\ \text{For NFA}:I=Imax/{\left(1+([\text{inh}]{/\text{IC}}_{50}\right)}^{nH}) \end{array}$$

where *I* is the normalized current amplitude induced by the agonist at concentration [A], [inh] – concentration of NFA, *Imax* is a maximal current induced at given cell, *n*_*H*_ is the Hill coefficient and EC_50_ or IC_50_ are the concentrations at which a half-maximum response was induced.

The fractional electrical distance from the external side of the membrane (δ) at which bound NFA blocked the current was calculated using the Woodhull equation:

$${\text{IC}}_{50}(\text{V})={\text{IC}}_{50}(0)\text{ exp }(-\delta \text{FV}/\text{RT}),$$

where IC_50_(V) is IC_50_ at the given membrane potential, IC_50_(0) – at 0 mV, V – membrane potential and F, R, T have their usual meanings. δ was determined from a plot of IC_50_ values against membrane potential.

For statistical analysis paired and unpaired *t*-tests were used. Data are represented as means ± SEM.

### Molecular modeling

#### General features of the model

The cryo-EM structure of the open state α1 GlyR (Du et al., 2015) (PDB code 3JAE) was used to create models of transmembrane parts of α1 and α2 GlyR channels and explore interactions of NFA with these models. We removed the extracellular region and focused on the central pore region, which is lined by five M2 helices. In the α2 GlyR model, five Gly254 residues at level 2′ were replaced with alanines. Each model also included 10 M1 and M3 helices. The 15-helix bundles of α1 and α2 GlyRs were optimized using the ZMM program (http://www.zmmsoft.com) and the Monte Carlo energy-minimization (MCM) protocol (Li and Scheraga, 1987) as described elsewhere (Bruhova et al., 2008; Garden and Zhorov, 2010). Molecular images were created using the PyMol Molecular Graphics System, Version 0.99rc6 (Schrödinger, LLC, New York, NY).

#### Niflumic acid geometry

In the X-ray structures of free NFA (Murthy and Vijayan, 1978) the molecule is virtually planar. In the available X-ray structures of proteins with NFA (Jabeen et al., 2005) (PDB codes 1TD7 and 2WM3) it is also nearly planar. We have built the ligand using a 2-fold torsional potential for the Ph-COO bond (benzoic acid) with the barrier height of 3 kcal/mol and minima at 90 and −90° (Lautenschlager and Brickmann, 1991). NFA adopted a nearly planar conformation with the intramolecular H-bond NH—O (Figure 1).

#### Pulling NFA through the pore

The interaction energy of NFA with the channel was computed as in Bruhova and Zhorov (2010). We pulled NFA from the cytoplasmic side to the extracellular side of the pore starting from the level below −2′ (Pro250) to level 20′ (Ala/Gly272) with the step of 0.5 Å. At each step, the nitrogen atom located between two rings of NFA (N1) was constrained to the plane, which is normal to the pore axis and crosses the axis at this level. To prevent “escape” of NFA from the pore, another constraint was used that allowed atom N1 to deviate up to 6 Å from the pore axis and imposed an energy penalty for larger deviations. At each level of the pore, the energy was MC-minimized until 100 consecutive energy minimizations did not decrease the best energy found at this level. To preclude large deviations of the model backbones from the X-ray templates and thus preserve the channel folding during docking of flexible ligand to the flexible protein, a set of distance constraints (pins), was imposed between matching alpha carbons in the X-ray structure and in the model. A pin constraint is a flat-bottom parabolic energy function that allows an atom (in this study, an alpha carbon) to deviate penalty-free up to 1 Å from the template and imposes a penalty of 10 kcal mol^−1^ Å^−1^ for larger deviations. The MC-minimization protocol optimized the system energy that included the penalty energy. In every MC-minimized structure, the energy of constrains was close to zero indicating that the ligand binding did not affect the protein folding in our models.

## Results

### Action of niflumic acid on α1 GlyRs

The NFA ability to modulate currents in different GlyR subtypes was determined using a whole-cell configuration of patch-clamp technique. GlyRs composed of different subunits were transiently expressed in the CHO cell line. First, we have examined the effect of varying concentrations of NFA on homomeric α1 GlyR at constant holding membrane potentials (V_hold_) of +30 and −30 mV using a “long” protocol of solutions application. Ionic currents were evoked by 30 μM of glycine alone (near EC_50_ concentration for α1 and α2 GlyRs, Maleeva et al., 2015) or mixed with different NFA concentrations. The inhibition was more pronounced at positive membrane potentials (Figure 2A). Thus, at V_hold_ of −30 mV, concentrations of NFA 30, 100 and 300 μM caused inhibition of α1GlyR-mediated currents by 3 ± 4, 16 ± 6, and 48 ± 9%, respectively (*n* = 7), while at V_hold_ of +30 mV currents were inhibited by 16 ± 7, 43 ± 11, and 75 ± 4%, respectively (*n* = 7).

To examine in detail the voltage dependence of GlyR block by NFA we have used a “ramp” protocol that allowed fast changes of the membrane potential from −80 to +80 mV (Figure 2B). Representative current-voltage dependence curves recorded during application of glycine alone or mixed with different concentrations of NFA are shown in Figure 2C. Glycine concentration of 30 μM (near EC_50_) produced an outwardly rectifying current due to the higher probability of the open-state α1 GlyR at positive potentials (Fucile et al., 1999). While NFA exhibited rather low affinity to α1 GlyR, especially at negative potentials, we have revealed significant (*p* < 0.01) voltage dependence of inhibition. Figure 2E shows that at −80 mV IC_50_ of NFA was 315 ± 30 μM, while at +80 mV it was 197 ± 18 μM (*n* = 10). The voltage dependence of inhibition suggests that NFA acts as an open channel blocker of α1 GlyRs.

To test this hypothesis we performed the same experiment with higher, near-saturating concentration of glycine (100 μM) that causes longer mean open time of GlyR channels. The potency of the channel block by NFA increased, particularly at positive potentials (*p* < 0.001). IC_50_ of NFA at −80 mV was 270 ± 26 μM, while at +80 mV about 3-fold decrease was observed: IC_50_ = 90±8 μM (*n* = 10) (Figures 2D,E). These results provide additional support to the suggestion that NFA inhibits α1 GlyR currents as an open channel blocker.

### Action of niflumic acid on α2 GlyRs

**Figure 3:**
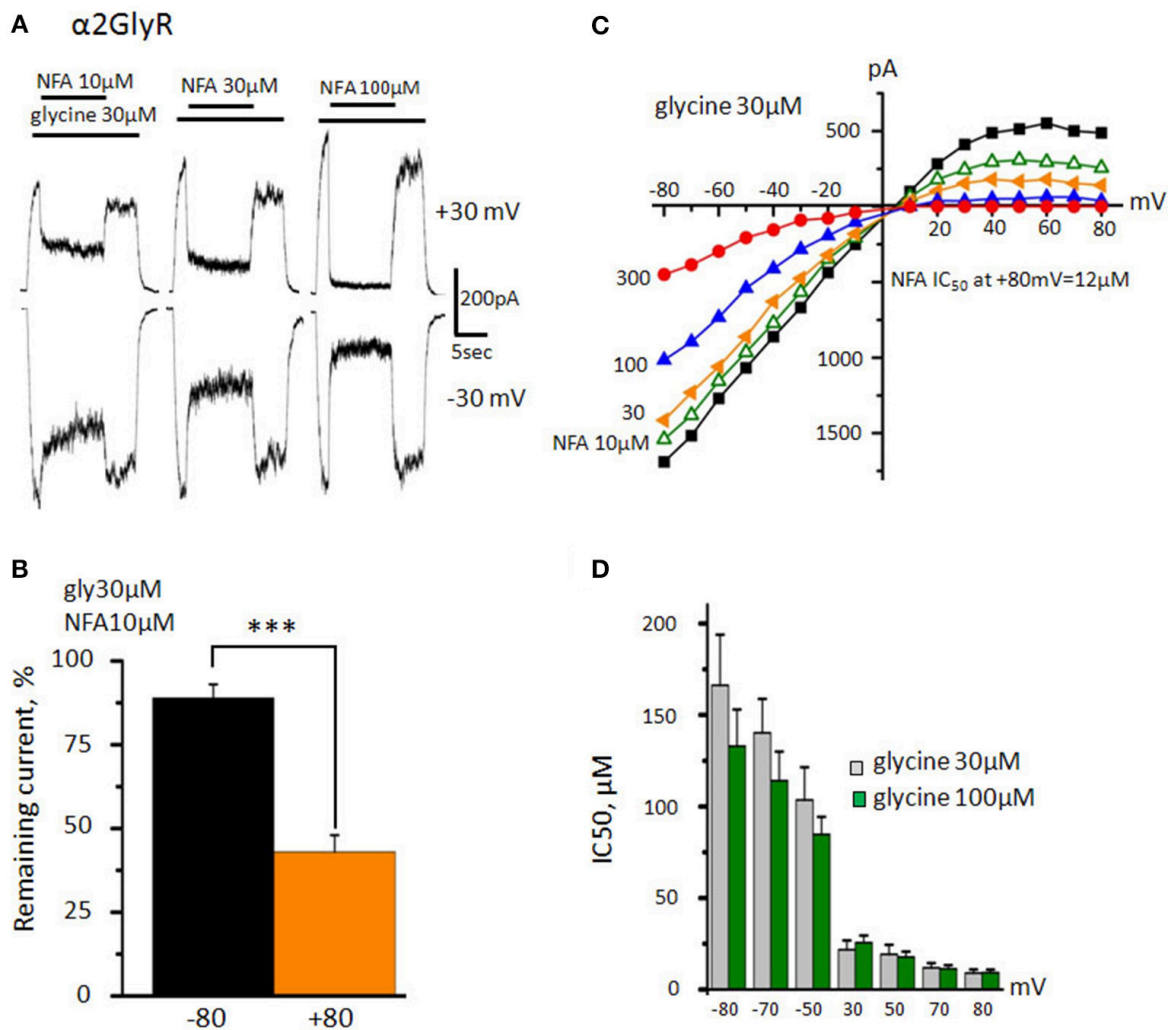
**Action of NFA on the α2 GlyR. (A)** Inhibition of glycine-induced currents (30 μM) by different concentrations of NFA (10, 30, and 100 μM). V_hold_ is +30 mV (upper traces) and −30 mV (bottom traces). **(B)** Percentage of the current that remained under application of 10 μM of NFA at +80 and −80 mV. Data from 5 cells. ^***^p < 0.001, paired *t*-test. **(C)** Representative current-voltage curves obtained in the presence of 30 μM glycine alone or in mixture with different concentrations of NFA (10 μM-green, 30-yellow, 100-blue, 300-red). **(D)** NFA IC_50_ at different potentials. Channels were activated by 30 μM (gray, *n* = 8) or 100 μM (green, *n* = 7) of glycine. Data represented as mean ± SEM.

Analysis of NFA action on α2 GlyR revealed two important peculiarities. Firstly, its inhibitory activity was much higher in comparison with that on α1 GlyR. Secondly, the voltage-dependence of inhibition was much more profound. Using a “long application” protocol we have demonstrated that NFA concentration as small as 10 μM inhibited currents by ~50% at MP of +30 mV (Figure 3A). The voltage dependence of α2 GlyR inhibition by NFA (10 μM) was also prominent: at MP = −80 mV glycine-evoked current comprised 89 ± 4% from the control, while at +80 mV it was as small as 43 ± 5% (*n* = 5) (Figure 3B).

To study in detail the voltage dependence of α2 GlyR block by NFA, we have used the same “ramp” protocol as we did for α1 GlyR. Unlike in α1 GlyRs, activation of α2 GlyRs by nonsaturating agonist concentration (30 μM) produced inwardly rectifying currents, suggesting that the open probability of α2 GlyR channel is higher at negative potentials. Another peculiarity of NFA action on α2 GlyRs was the strong voltage dependence of inhibition (Figure 3C). Thus, at −80 mV IC_50_ of NFA was 166 ± 28 μM, while at +80 mV it decreased nearly 20 times, to 9 ± 2 μM (*n* = 8). Contrary to α1 GlyRs, the potency of NFA block did not increase further with elevation of glycine concentration (Figure 3D; *p* > 0.05). Currents induced by 100 μM of glycine were inhibited by NFA with IC_50_ of 133 ± 20 μM at −80 mV and 9 ± 2 μM at +80 mV (*n* = 7).

The strong voltage dependence of the block suggests that NFA is an open channel blocker interacting with residues located deeply in the pore.

This suggestion was confirmed by results of single channel recordings performed in outside-out patches from cells expressing α2 GlyRs in the presence of glycine or mixture of glycine with NFA (Supplementary Figure 1). In control conditions, application of 5 μM glycine at V_hold_ −30 mV induced single channel openings (mean amplitude 2,8 pA) with rare short closings (Supplementary Figure 1A). Addition of 30 μM NFA transferred rectangular single channel pulses into bursts with high frequency interburst flickering (Supplementary Figure 1B). The effect became much more profound at elevation of the NFA concentration to 100 μM (Supplementary Figure 1C). This phenomenology is similar to earlier described phenomenology of channel blockers, for instance, action of local anesthetics on single acetylcholine-receptor channels (Neher and Steinbach, 1978) or flickering mode of NMDA receptors channel block by Mg^++^ (Nowak et al., 1984) or amantadine (Blanpied et al., 2005).

### Action of niflumic acid on α3 GlyRs

Since NFA has shown distinct profiles of interaction with α1 and α2 GlyRs, we decided to explore its action on α3 GlyR with the aim to determine the critical amino acids for the blocker action. Notably, the TM2 helix in α3 GlyR is the same as in α2 GlyR. Both possess alanine in position 2′ (**Figure 9A**) implying that NFA may block α3 GlyR as strongly as α2 GlyR and stronger than α1 GlyR, which has glycine at position 2′.

**Figure 4:**
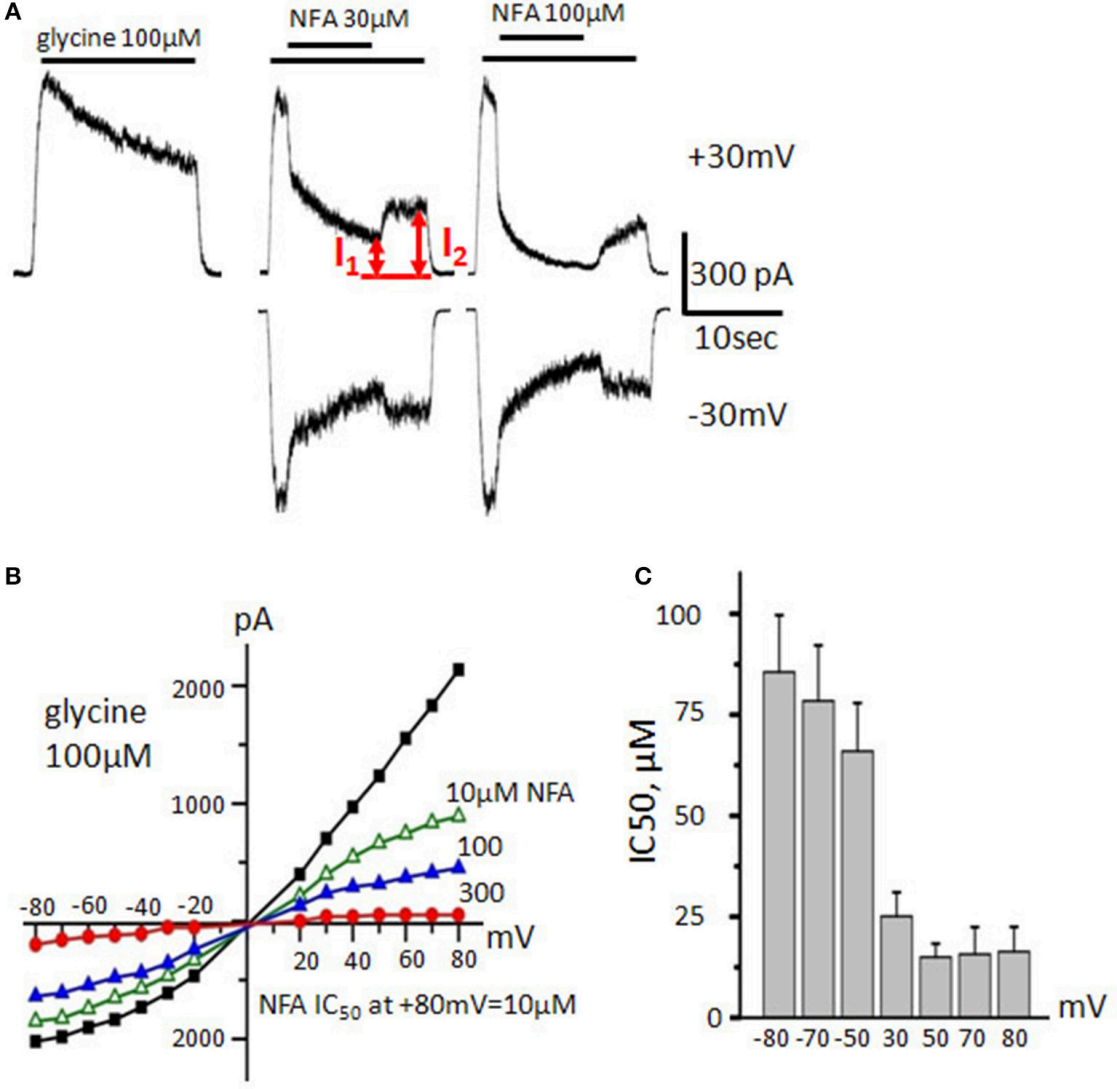
**Action of NFA on α3 GlyRs. (A)** Traces of the control glycine–induced current (100 μM, left trace) and its inhibition by different concentrations of NFA (30 and 100 μM, middle and right traces). V_hold_ is +30 mV (upper traces) and −30 mV (bottom traces). I_1_ and I_2_ are amplitudes of currents used to calculate the degree of inhibition. **(B)** Representative current-voltage relationships recorded during application of glycine (100 μM) alone or in presence of different concentrations of NFA. **(C)** NFA IC_50_ at different membrane potentials. Currents were estimated in the presence of 100 μM glycine. Data from 7 cells, represented as mean ± SEM.

Indeed, during “long application” protocol, α3 GlyR-mediated currents induced by 100 μM of glycine (near EC_50_ concentration for α3 GlyRs, Maleeva et al., 2015) were blocked by NFA with the higher potency than currents mediated by α1 GlyRs (Figure 4A). 30 μM of NFA inhibited α3 GlyR-mediated currents by 32 ± 4% at −30 mV and by 62 ± 5% at +30 mV, while 300 μM NFA at −30 mV inhibited ionic currents by 86 ± 5% and by 90 ± 2% at +30 mV (*n* = 7). It is well documented that α3 GlyRs rapidly desensitize (Nikolic et al., 1998) and this complicates accurate estimation of the NFA blocking potency. Thus, over all the cells percentage of inhibition was measured as the ratio of the current amplitudes at the maximal inhibition (**I**_1_) and after washout of NFA, when the glycine-induced current reaches the quasi-stationary level (**I**_2_, Figure 4A).

Interestingly, the current/voltage dependence recorded during application of 100 μM of glycine demonstrated the outward rectification, similar to that observed for α1 GlyRs. Like for both α1 and α2 GlyRs, the efficiency of α3 GlyRs block by NFA was higher at positive potentials (Figures 4B,C). At MP of −80 mV and +80 mV, IC_50_ of NFA was 86 ± 14 and 16 ± 6 μM, respectively (*n* = 7).

Thus, the sensitivity of α3 GlyRs to NFA is higher than that of α1 GlyRs and is rather close to that of α2 GlyRs.

### Niflumic acid action on the α1 GlyR mutant G254A

**Figure 5:**
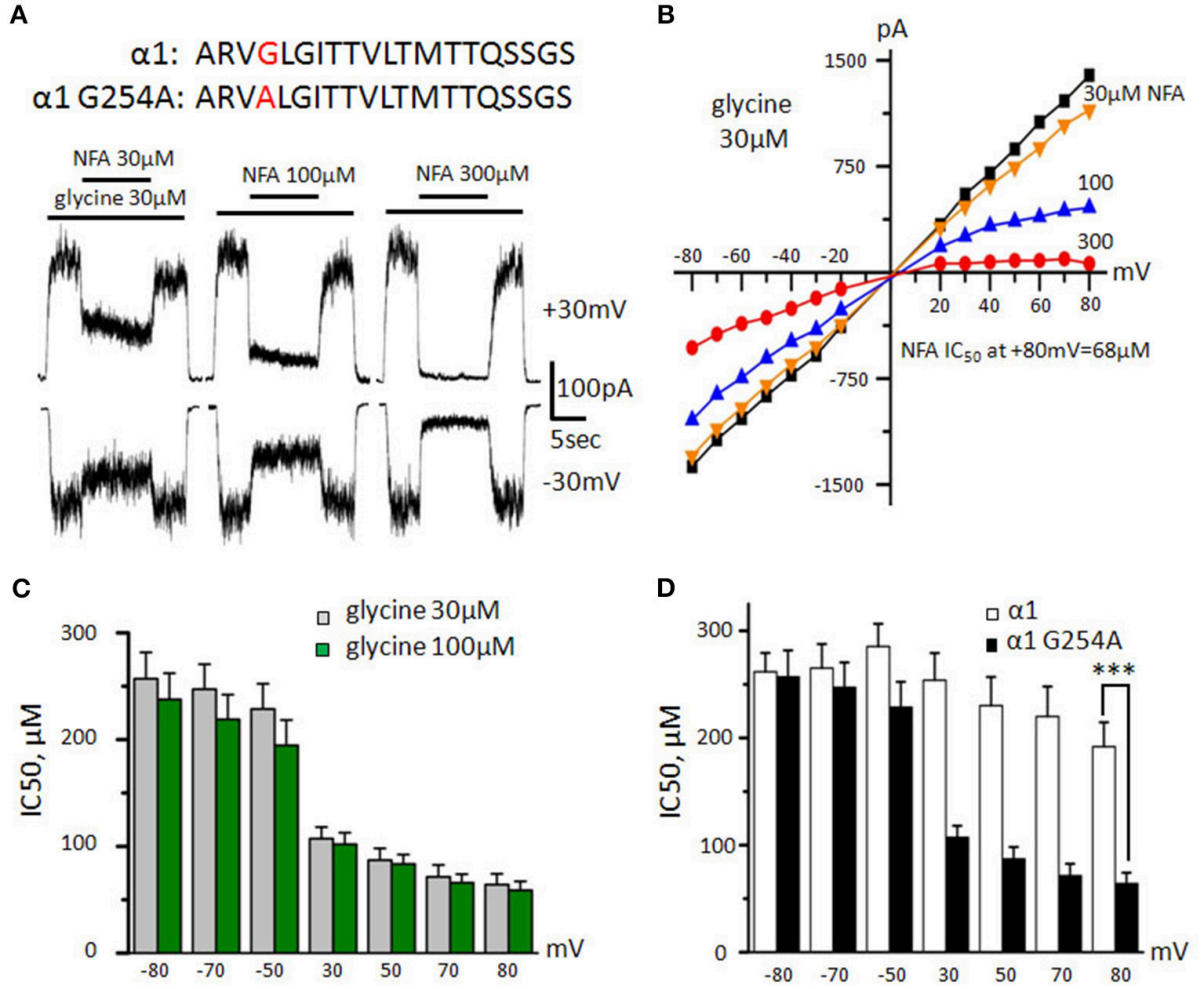
**Action of NFA on G254A-α1 GlyR. (A)** Amino acid sequences of the TM2 domain of α1 wild type and mutant subunits (mutated residue is highlighted by red) and recordings of inhibition of glycine-induced currents (30 μM) by different concentrations of NFA (30, 100, and 300 μM); V_hold_ is +30 mV (upper traces) and −30 mV (bottom traces). **(B)** Representative current-voltage relationships recorded during application of glycine (30 μM) alone or in the presence of different concentrations of NFA. **(C)** NFA IC_50_ at different potentials, currents were induced by application of 30 μM (gray, *n* = 13) and 100 μM (green, *n* = 8) of glycine. **(D)** Comparison of NFA sensitivities of α1 GlyR wildtype (white, *n* = 8) and G254A-α1 GlyR (black, *n* = 13). Currents were induced by 30 μM glycine, ^***^p < 0.001, unpaired *t*-test. Data represented as mean ± SEM.

Taking into account the voltage dependence and presumably pore-blocking mechanism of NFA action, we suggested that amino acids, which are crucial for the interaction of NFA with GlyRs, are located in the pore-lining helices. The TM2 helices are highly conserved between different α subunits of GlyRs and differ only at position 2′ where α1 subunit has glycine, while α2 and α3 subunits have alanine (**Figure 9A**). We suggested that this difference is the main determinant of the distinct profiles of inhibition of different GlyRs by NFA. To test this hypothesis, we performed a point mutation at position 254 of the α1 subunit exchanging glycine for alanine (Figure 5A). We expected to convert low NFA sensitivity of the α1 GlyR to high sensitivity, characteristic for α2 and α3 GlyRs.

The dose/response relationships showed that the G254A-α1 GlyR mutant sensitivity to glycine is similar to that of the wild-type α1 GlyR: the EC_50_ was 34 ± 6 μM (*n* = 6, data not shown), which agrees with previous data (Shan et al., 2001). At the “long application” protocol, NFA relatively weakly inhibited G254A-α1 GlyR mediated currents induced by 30 μM glycine, but its potency was higher than that in the wild-type α1 GlyRs (Figure 5A). At V_hold_ of −30 mV, 30, 100, and 300 μM of NFA inhibited G254A-α1 GlyR mediated currents by 22 ± 4, 34 ± 6, and 52 ± 11%, respectively (*n* = 6), while at V_hold_ of +30 mV, the same NFA concentrations inhibited currents by 23 ± 7, 43 ± 10, and 76 ± 4%, respectively (*n* = 7).

Using the “ramp” protocol, we found the above effect to be more pronounced at higher potentials (Figure 5B). Notably, at +80 mV currents induced by 30 μM of glycine were inhibited by NFA with IC_50_ of 64 ± 10 μM (*n* = 13; Figure 5D), which was significantly lower (*p* < 0.001) than for the wild-type α1 GlyR (192 ± 23 μM, *n* = 8). However, at negative potentials sensitivity of the G254A-α1 GlyR was close to that of wild type α1 GlyR and comprised 257 ± 25 μM (*n* = 13). With the increase of agonist concentration, the strength of the block did not increase significantly and comprised 58 ± 8 μM (*n* = 8) at MP = +80 mV.

Importantly, the NFA blocking potency for G254A-α1 GlyR was lower than that for α2 and α3 GlyRs. This suggests that amino acids beyond the TM2 helices affect the NFA action, likely by an allosteric mechanism.

### Woodhull analysis of voltage dependence of the GlyR channel block by NFA

**Figure 6:**
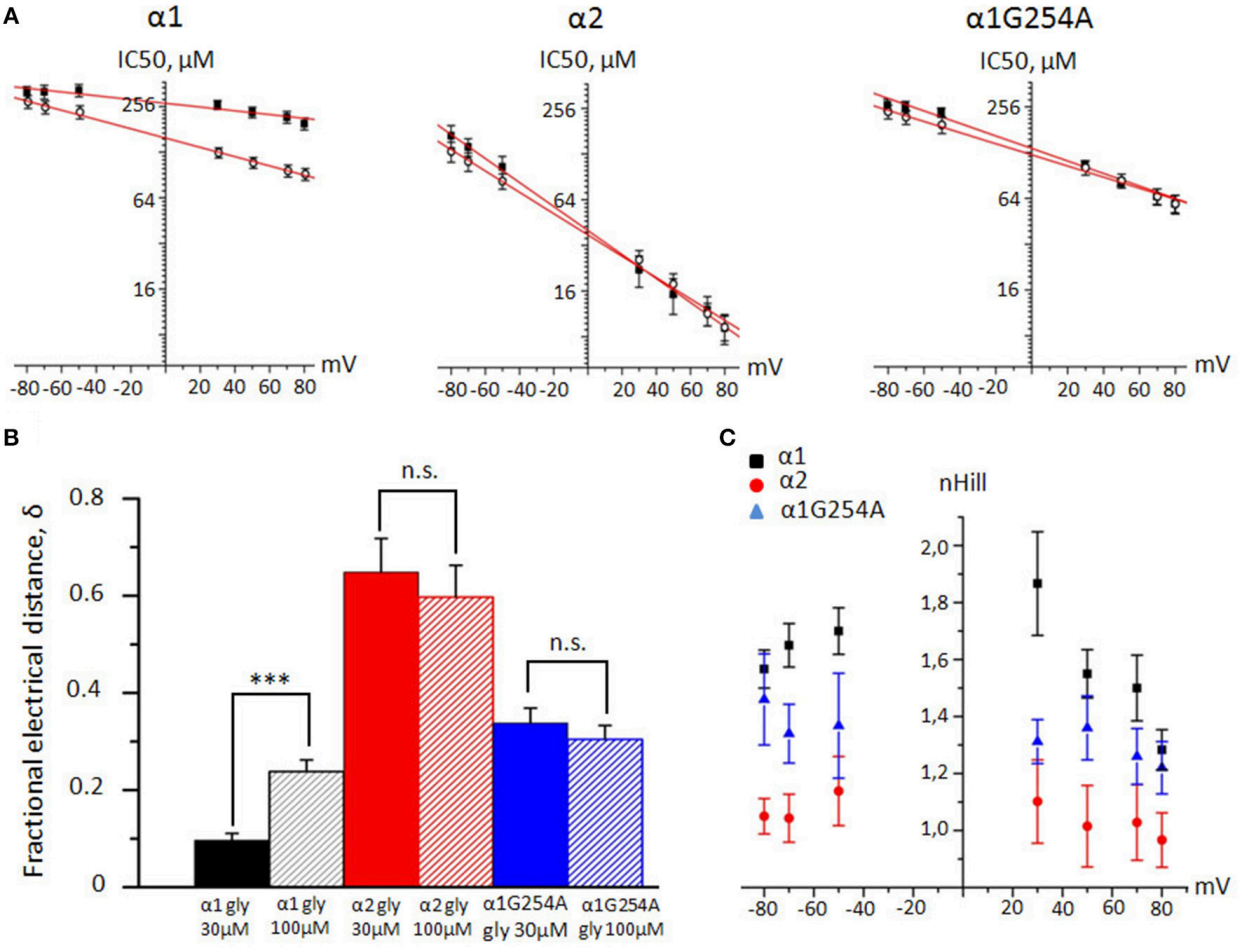
**Woodhull and cooperativity analysis of NFA interaction with α1, α2, and α1 G254A GlyRs. (A)** Linear fit of the cumulative IC_50_ values plotted against membrane potential for α1, α2 and G254A-α1 GlyRs. Currents were evoked by 30 μM of glycine (filled squares) or by 100 μM of glycine (empty circles). **(B)** Mean fractional depth of NFA binding in the pore (δ) for α1 (black column), α2 (red) and G254A-α1 (blue) GlyRs. Currents were evoked by 30 (filled columns) and 100 μM (shaded) of glycine. Data from 6–10 cells. ^***^p < 0.001, unpaired *t*-test; n.s. – nonsignificant. **(C)** Cumulative n_H_ values of NFA interaction with α1 (black squares, *n* = 6), α2 (red circles, *n* = 6) and G254A-α1 (blue triangles, *n* = 5) GlyRs plotted against membrane potentials. Currents were evoked by 30 μM of glycine. Data represented as mean ± SEM.

To estimate the fractional depth of the NFA binding sites in the pore with respect to the external membrane surface, we used classical Woodhull analysis developed to describe the hydrogen block of sodium channels (Woodhull, 1973). We have plotted NFA IC_50_ values against membrane potential for α1, α2, and G254A-α1 GlyRs at glycine concentrations of 30 and 100 μM (Figure 6A). The calculated values of δ correspond to the percentage of the transmembrane electric field that is sensed by the bound NFA (Figure 6B). For the NFA block of α1 GlyR, mean δ values at 30 and 100 μM of glycine were 0.16 ± 0.03 (*n* = 7) and 0.26 ± 0.02 (*n* = 10), respectively. The NFA binding site in α2 GlyR was found to be significantly deeper. With 30 and 100 μM of glycine, the sensed electric field (δ) increased to 0.65 ± 0.07 and 0.63 ± 0.07, respectively (*n* = 7, *p* < 0.001). The NFA binding site in the G254A-α1 GlyR was situated between the sites in the α1 and α2 GlyRs, at the level of ~ 30% from the extracellular side at both examined agonist concentrations (Table 1).

**Table 1:** **Fractional electrical distance (δ) from the extracellular side of the membrane at which NFA blocks the pore in α1, α2 and G254A-α1 GlyRs**.

**Glycine concentration**	**α1 GlyR**	**α2 GlyR**	**G254A-α1 GlyR**
30 μM	0.16 ± 0.03 (*n* = 7)	0.65 ± 0.07 (*n* = 7)	0.37 ± 0.04 (*n* = 9)
100 μM	0.26 ± 0.02 (*n* = 10)	0.63 ± 0.07 (*n* = 7)	0.30 ± 0.04 (*n* = 7)

*The data are represented as mean ± SEM*.

### Hill coefficient of NFA/GlyR interaction

To estimate cooperativity of NFA inhibition of GlyRs, we determined the Hill coefficient (n_H_) for α1, α2, and G254A-α1 GlyRs. The amplitude of glycine-evoked currents at different potentials was plotted against the NFA concentration and fitted with the Hill equation. At positive membrane potentials, n_H_ decreased with the membrane depolarization for the three GlyRs (Table 2 and Figure 6C). For α1 and α2 GlyRs n_H_ was significantly different at all the tested membrane potentials (−80 mV, −70 mV *p* < 0.001; −50 – +50 mV *p* < 0.01; 70, 80 mV *p* < 0.05), and for the G254A-α1 GlyR n_H_ values were between those for α1 and α2 GlyRs. Concentration dependencies of NFA action on different GlyR subunits are presented in the Supplementary Figure 2.

**Table 2:** **Hill coefficient (n_H_) of NFA interaction with α1, α2, and G254A-α1 GlyRs at different membrane potentials**.

**Membrane potential (mV)**	**α1 GlyR (*n* = 6)**	**α2 GlyR (*n* = 6)**	**G254A-α1 GlyR (*n* = 5)**
−80	1.56 ± 0.07	1.05 ± 0.06	1.46 ± 0.16
−70	1.65 ± 0.08	1.04 ± 0.08	1.34 ± 0.1
−50	1.7 ± 0.08	1.14 ± 0.12	1.37 ± 0.18
30	1.87 ± 0.02	1.1 ± 0.14	1.3 ± 0.08
50	1.55 ± 0.08	1 ± 0.14	1.36 ± 0.11
70	1.5 ± 0.11	1 ± 0.13	1.26 ± 0.1
80	1.28 ± 0.07	0.97 ± 0.09	1.22 ± 0.09

*Data are represented as mean ± SEM*.

These data suggest that the cooperativity of NFA block strongly depends on the GlyR subtype.

### Action of niflumic acid on α1β and α2β GlyRs

In the adult CNS of vertebrates, the predominant GlyR subtype is heteromeric α1β (Lynch, 2004). Several inhibitors, e.g., picrotoxin (Pribilla et al., 1992; Wang et al., 2006) and ginkgolides (Kondratskaya et al., 2005) have different affinity in heteromeric and homomeric GlyRs. To estimate the NFA sensitivity to heteromeric GlyRs we studied its interaction with α1β and α2β GlyRs. The concentration of the agonist was chosen according to the previous studies, demonstrating that 30 μM of glycine is below EC_50_ for α1β and α2β glycine receptors (Rundstrom et al., 1994; Miller et al., 2005).

**Figure 7:**
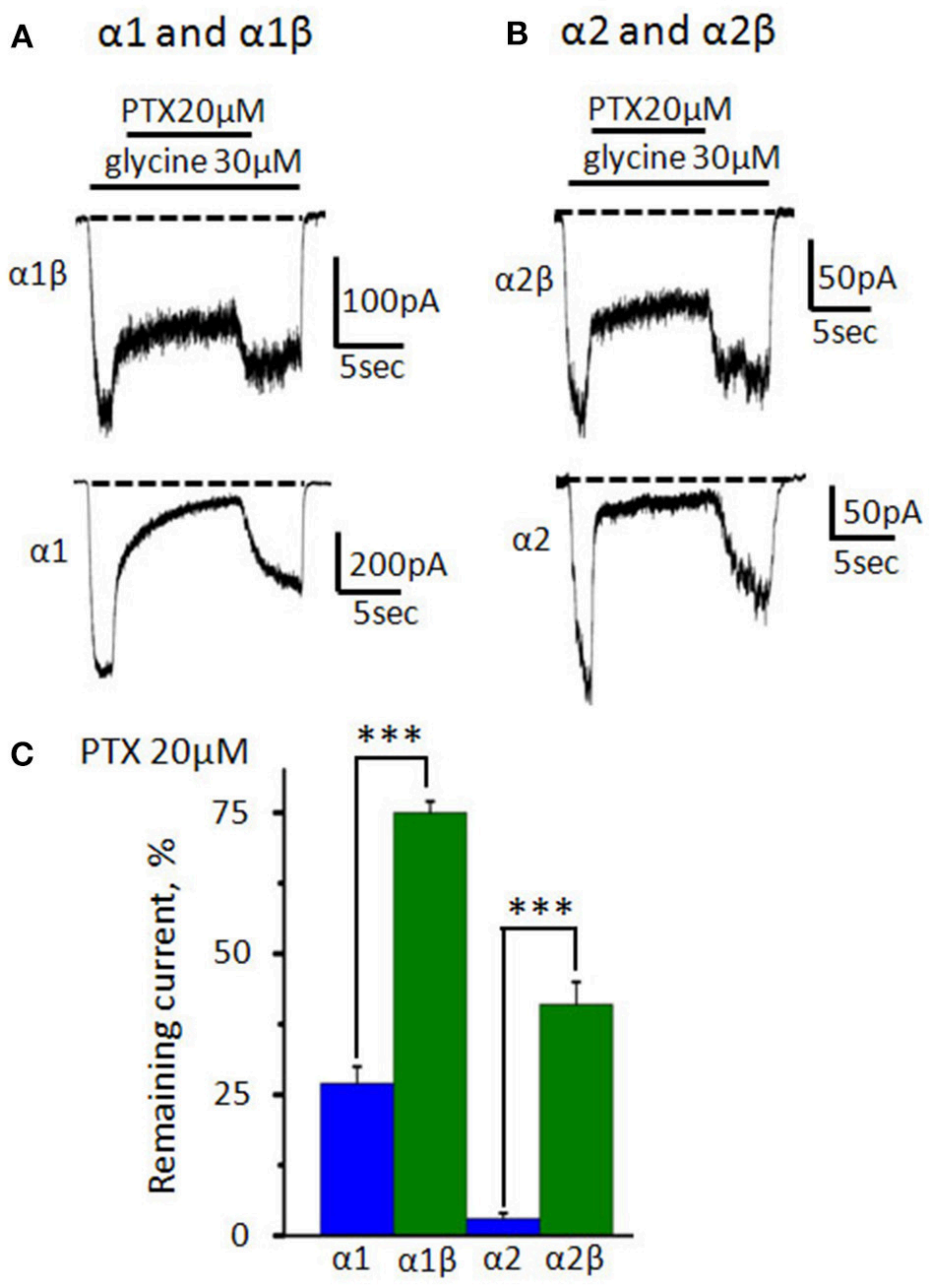
**Inhibition of homomeric and heteromeric receptors by picrotoxin. (A)** The action of PTX (20 μM) on currents mediated by α1β (upper trace) and α1 GlyRs (bottom trace). The currents were evoked by 30 μM of glycine; V_hold_ = −30 mV. **(B)** The action of PTX (20 μM) on currents mediated by α2β (upper trace) and α2GlyRs (bottom trace). The currents were evoked by 30 μM of glycine; V_hold_ = −30 mV. For clarity, dotted lines show the current levels in the absence of the agonist. **(C)** Percentage of the remained current from control under application of PTX (20 μM) for α1 (*n* = 12), α1β (*n* = 15), α2 (*n* = 9), and α2β (*n* = 7) GlyRs (homomeric receptors—blue, heteromeric—green). Data represented as mean ± SEM, ^***^p < 0.001, unpaired *t*-test.

To prove formation of functional heteromeric GlyRs, we implemented a widely used picrotoxin (PTX) test: PTX blocks αXβ GlyRs much weaker than homomeric αX GlyRs (Pribilla et al., 1992; Shan et al., 2001). In our preparations, under application of 20 μM of PTX amplitudes of α1 and α2 GlyR -mediated currents were, respectively, 27 ± 3% (*n* = 12) and 3 ± 1% (*n* = 9) of the control. Heteromeric GlyRs were inhibited much weaker: the current in α1β GlyR was 75 ± 2% (*n* = 15) and in α2β GlyR it was 41 ± 4% (*n* = 7) from the control (Figures 7A–C).

**Figure 8:**
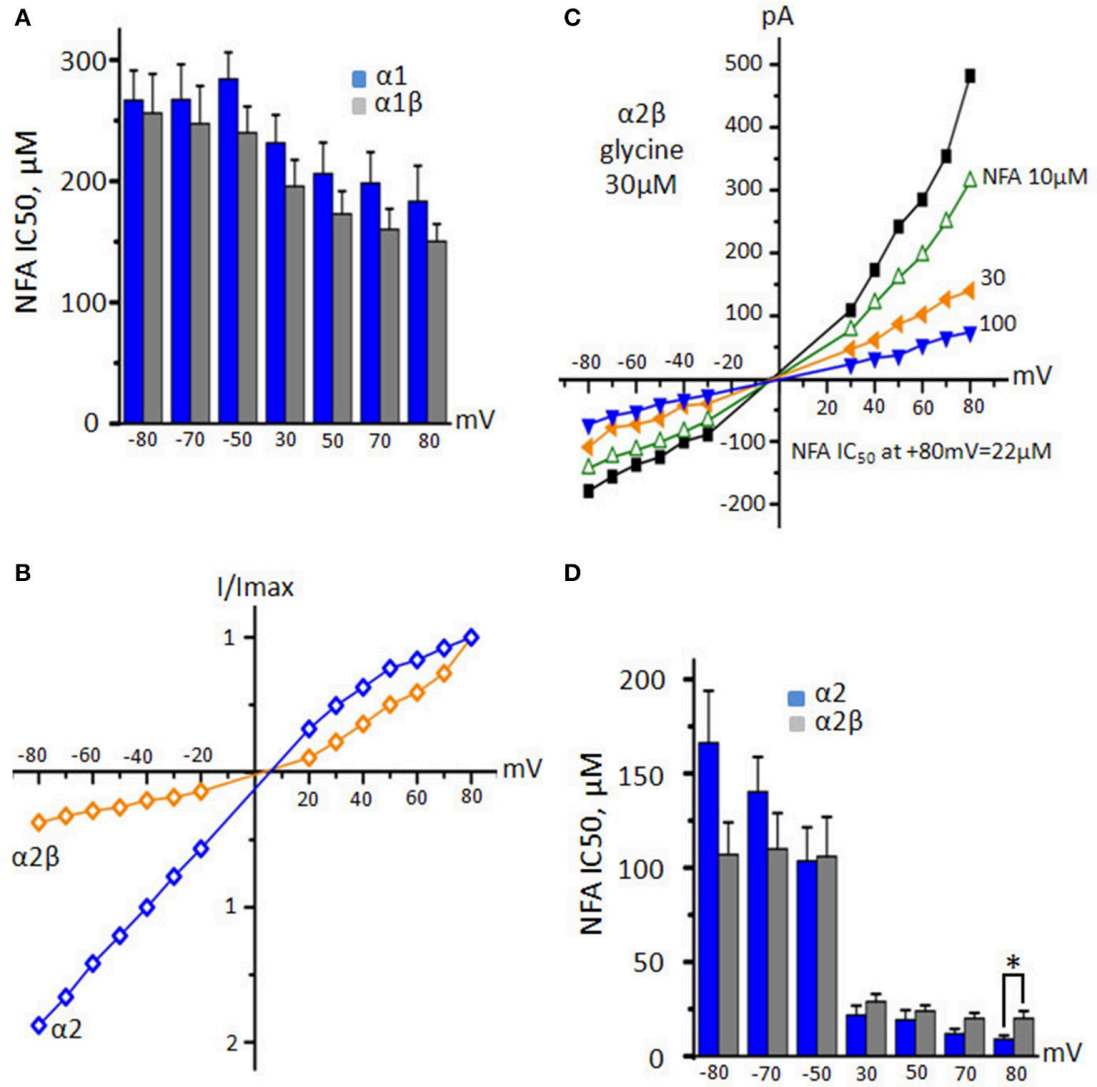
**Action of NFA on heteromeric α1β and α2β GlyRs. (A)** NFA IC_50_ values for α1 (blue, *n* = 10) and α1β (gray, *n* = 5) GlyRs at different potentials. Currents were induced by application of 30 μM glycine. **(B)** Normalized representation of current-voltage relations from two cells expressing α2 (blue) and α2β (orange) GlyRs at application 30 μM glycine. **(C)** Representative I/V curves from one cell recorded for α2β GlyR during application of 30 μM of glycine alone or in the presence of different concentrations of NFA. **(D)** Comparison of NFA IC_50_ for α2 (blue, *n* = 7) and α2β (gray, *n* = 5) GlyRs at different potentials. Currents were induced by application of 30 μM of glycine. Data represented as mean ± SEM, ^*^p < 0.05, unpaired *t*-test.

Estimation of NFA IC_50_ at different membrane potentials has shown that incorporation of the β subunit does not change significantly (*p* > 0.05) the sensitivity of α1 GlyR to NFA: at +80 mV IC_50_ of NFA was 150 ± 14 μM (*n* = 5) (Figure 8A). Analysis of I/V curves also revealed a similarity to α1 GlyRs: currents mediated by α1β GlyRs (30 μM of glycine) were outwardly rectifying (data not shown).

Surprisingly, α2β GlyRs demonstrated the outwardly rectifying currents, contrary to inwardly rectifying α2 GlyRs (Figures 8B,C). In comparison to α2 GlyRs NFA stronger inhibited heteromeric α2β receptors at negative potentials being less effective at positive ones. This resulted in slightly weaker voltage dependence of the block vs. homomeric GlyRs: at −80 and +80 mV, IC_50_ was 107 ± 37 and 20 ± 8 μM, respectively (*n* = 5, Figure 8D). In general, the β subunit does not have a strong impact on the interaction of NFA with GlyRs.

### Computational search for possible NFA binding sites in the pore of GlyRs

A systematic search for possible binding sites of NFA in different regions of the large GlyR proteins was beyond goals of this study. Here we focused on the pore region where NFA is likely to bind. Several arguments support this proposition. First, the voltage-dependence of the NFA action (Figures 2–6) strongly suggests that the compound binds within the membrane. Second, the anionic NFA molecules would enjoy favorable electrostatic interactions within the anion-selective pore. Third, results of single channels experiments demonstrated dramatical increase of the open state fluctuations caused by NFA application. Fourth, upon the channel activation, the α2 GlyR appears to open for a longer time than α1 GlyR (Takahashi et al., 1992; Morales et al., 1994). The longer openings would give NFA molecules more chances to reach their binding site(s) within the pore. This may explain why α2 GlyR is more sensitive to the NFA block than α1 GlyR.

**Figure 9:**
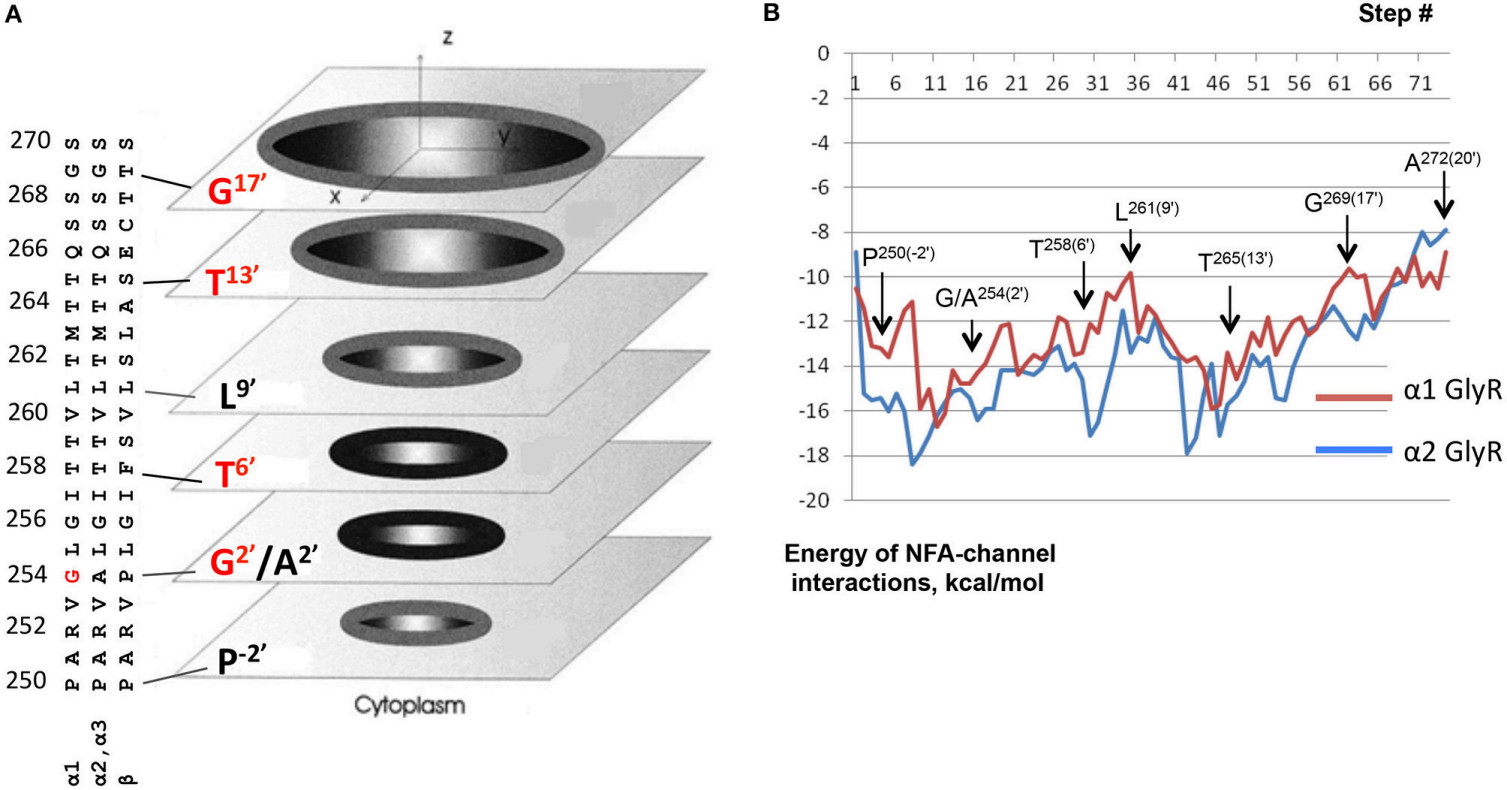
**Monte Carlo energy minimized profiles of NFA pulled through the pore models of α1 and α2 GlyRs. (A)** A schematic view of the rings of the pore-facing residues at different levels of the pore. Relative dimensions of the rings and distances between the planes do not exactly match the computational models. Amino acid sequences of the pore-lining TM2 helices in α1, α2/α3, and β subunits left to the scheme should be read from the bottom to the top. The numbering of amino acids is used as for human α1 GlyR starting with the N-terminal, i.e., without the signal peptide sequences (Grenningloh et al., 1990), as was used previously (David-Watine et al., 1999; Imboden et al., 2001; Breitinger et al., 2004). Note hydrophilic rings Thr^265(13′)^ and Thr^258(6′)^ above and below the hydrophobic ring Leu^261(9′)^. Rings Gly^254(2′)^ in α1 GlyRs and Gly^269(17′)^ are indicated as hydrophilic ones because their hydrophilic backbone atoms are exposed to the pore. **(B)** Interaction energy of NFA with the α1 and α2 GlyR models plotted against position of the NFA central nitrogen atom (N1, see Figure 1B). At each level of the pore, atom N1 was constrained to a plane, which is normal to the pore axis. During the MCM search for energetically preferable orientation of NFA at the given level, atom N1 was free to move within this plane and NFA molecule was free to rotate around N1. The arrows indicate positions of the N1 atom at the levels of pore-facing residues. Note that at some orientations the NFA molecule may extend along the pore up to 13 Å (Figure 1B) and thus interact with the pore-facing residues below and above the level where the N1 atom was constrained. For example, at level 2′, the α2 GlyR channel contains five pore-facing alanine residues, which are more attractive to the NFA molecule than five Gly2′ residues in the α1 GlyR channel. However, even when the N1 atom is constrained at level 6′, one part of the NFA molecule could interact with residues at level 2′ and another part with residues at level 9′ (Figure 10D).

**Figure 10:**
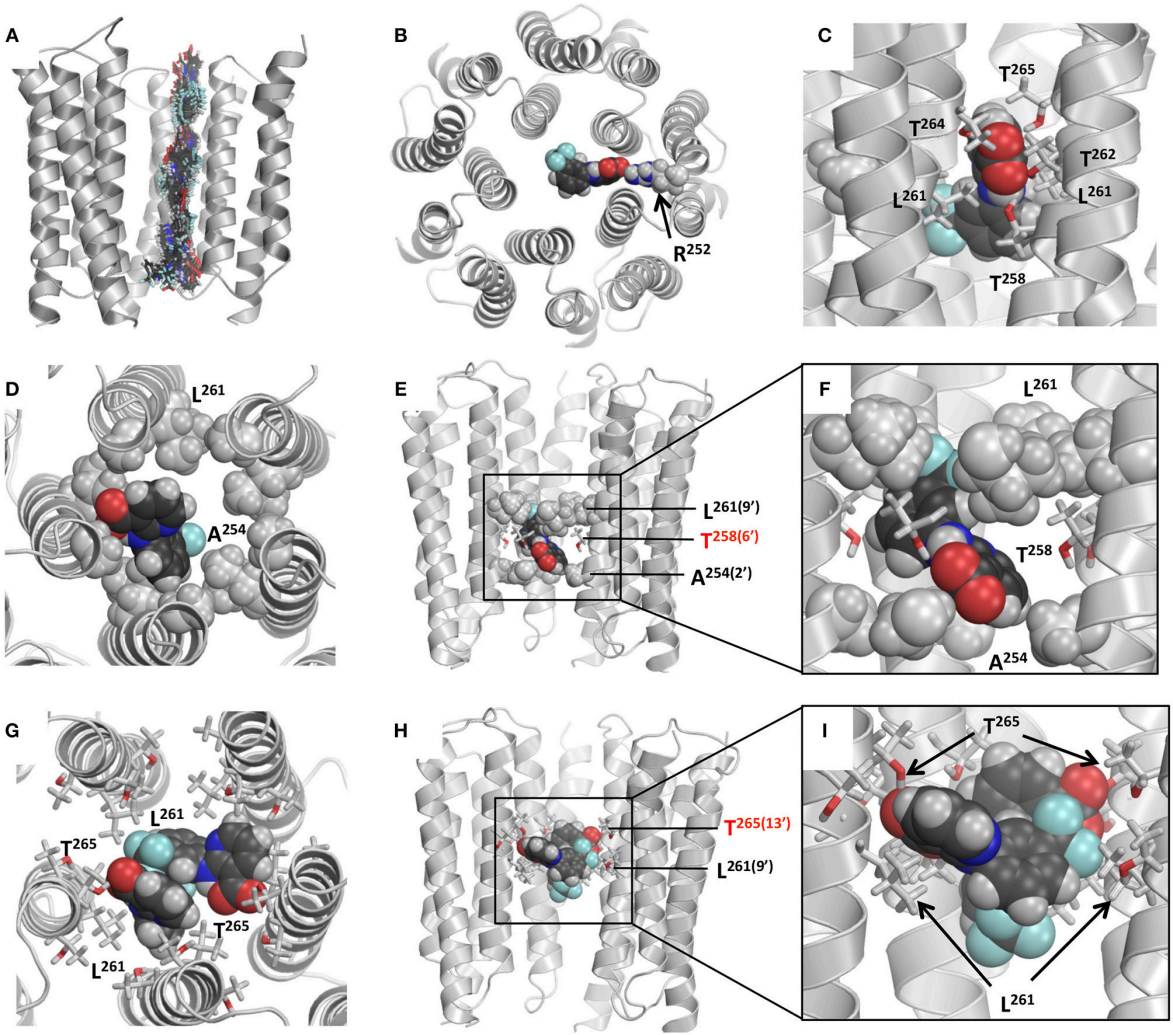
**Models of NFA binding in the GlyRs. (A)** Superposition of snapshots with NFA at different levels of the α2 GlyR model. Front subunit is removed for clarity. At each level, the energy is MC-minimizes as described in Materials and Methods. **(B)** Cytoplasmic view at α2 GlyR with the NFA molecule electrostatically attracted to Arg252. Due to this attraction both α1GlyR-NFA and α2 GlyR-NFA trajectories have deep minima at level −2′. **(C)** Side view of the α1 GlyR model with NFA at level 9′, which corresponds to the energy maxima in Figure 9. At this level NFA squeezes through the pore constriction formed by the Leu^261(9′)^ residues, but due to deep penetration of the NFA carboxylic group between two M2 helices, it may accept up to four H-bonds from Thr^258(6′)^, Thr^262^, Thr^264^ and Thr^265(13′)^. **(D–F)** Intracellular, side, and enlarged side views of the α2 GlyR model with atom N1 of NFA bound level 6′, where a minimum is seen at the energy profile (Figure 9B). The front subunit is removed for clarity at **(E,F)**. NFA accepts an H-bond from Thr^258(6′)^ and forms hydrophobic contacts with the rings of Ala^254(2′)^ and Leu^261(9′)^ residues by the trifluoromethyl-aryl and pyridine groups, respectively. **(G–I)** Extracellular, side, and enlarged side views of the α1 GlyR model two NFA molecules bound above the Leu^261(9′)^ ring. The front subunit is removed for clarity at **(H,I)**. Both NFA molecules accept H-bonds from Thr^265(13′)^, and form hydrophobic contacts with each other and Leu^261(9′)^ residues.

Figure 9B shows computed energy (kcal/mol) of NFA interaction with α1 and α2 GlyR models and Figure 10A shows superposition of 74 snapshots with NFA at different levels of the α2 GlyR pore. The α1 and α2 GlyR models differ only at level 2′ where they have, respectively, the rings of Gly254 and Ala254 residues, but the computed energy values are rather different at each level (Figure 9B). The cause is the NFA molecule size (~13 Å between most remote atoms, see Figure 1B), which is bigger than, e.g., the distance of 5.8 Å between C^β^ atoms of the same TM2 helix at levels 2′ and 6′. Since the preferable orientation of NFA in the pore is unknown, the NFA molecule was allowed to rotate during MC-minimizations at each step. As a result, once the energetically favorable orientation was found at some level, the probability to find the energetically better orientation at the same level become small. A bottom line from a large number of independent runs with different seed random numbers might yield smoother trajectories, but such computations, which would require large computational resources, were beyond goals of our study.

The NFA interaction energy with the α1 and α2 GlyR models differs not only around level 2′, but also at the levels where the pore-facing residues are identical. At most of the levels, the energy difference between the two models does not exceed 2 kcal/mol, but it reaches 5 kcal/mol at level 6′ (Thr258). Despite the differences, the two trajectories have several common features as described below. First, there are rather deep minima at the cytoplasmic side of the pore, which are due to electrostatic interactions between NFA and Arg252 (Figure 10B). Second, there are energy minima at level 6′, which are due to H-bonds between NFA and Thr258 residues and hydrophobic interactions of NFA with the intracellularly-oriented side of the Leu261 ring. Third, there are energy minima at level 13′, which are due to H-bonds between NFA and Thr265 and favorable hydrophobic interactions of NFA with the extracellularly-oriented side of the Leu261 ring. Fourth, there are energy maxima at level 9′, which are due to certain repulsion between NFA and Leu261 residues that form the open pore constriction. However, even at this level the NFA-channel energy remains favorable (around −10 kcal/mol), partially because the ligand carboxylic group can accept up to four H-bonds from Thr258, Thr264, and Thr265 (Figure 10C). Fifth, relatively large energies at the extracellular part of the pore (levels 17′ to 20′) are due to two reasons. (i) The pore at these levels is too wide and NFA cannot simultaneously interact with three helices as it does at lower levels. (ii) Levels 13′ and 17′ are both hydrophilic (Figure 9A) and predominantly hydrophobic NFA cannot form multiple favorable contacts with the pore-facing residues.

Based on these results we suggest that there are more than one site along the pore where NFA can bind and block the ion permeation. The most likely locations of the NFA binding sites are at the opposite sides of the Leu261 ring, which forms the open pore constriction. And the most likely mechanism of the current block is the combination of the hydrophobic and steric block: NFA exposes its hydrophobic groups to the pore lumen, binds above and/or below the Leu261 ring and thus precludes permeation of the hydrated chloride ion. This block may be enforced by the electrostatic repulsion of the negatively charged carboxylate group of NFA and negatively charged chloride ions.

Our data on the Hill coefficient and the fractional electrical distance of the NFA binding site suggest two peculiarities of NFA action on GlyRs. Firstly, two NFA molecules preferably block permeation through the α1 GlyR channel, whereas the α2 GlyR channel is blocked by a single NFA molecule. Secondly, the NFA binding site in the α2 GlyR is located deeper than in the α1 GlyR. These experimental data provide important constraints to further elaborate models of NFA-bound α1 and α2 GlyRs.

The α2 GlyR block by a single NFA molecule bound deeply inside the pore can be illustrated by the binding mode found without constraints (Figures 10D–F). In this mode, NFA binds between hydrophobic rings Ala254 and Leu261 and accepts an H-bond from Thr258. This and similar binding modes collected in the MC-minimizations correspond to the energy minima at the left part of the energy profile (Figure 9B).

The energy profile of NFA in the α1 GlyR shows that NFA-channel interactions at levels −2′ to 6′ are energetically less preferable than in the α2 GlyR. A reason is the ring of Gly254 residues in α1 GlyR, which does not form as favorable van der Walls and hydrophobic contacts with NFA as the ring of Ala254 residues in α2 GlyR does. In particular, water molecules that hydrate the pore-exposed hydrophilic backbone atoms of Gly254 residues would destabilize binding of the hydrophobic NFA.

A complex α1 GlyR with a single NFA bound above the ring of Leu261 residues, at level 13′ (Thr265) corresponds to the wide energy minima (Figure 9B). We docked the second NFA molecules to this complex and MC-minimizing the energy. The pore easily accommodated two NFA molecules at this level and calculations predicted several possible low-energy binding modes of the two ligands. One of these is shown at Figures 10G–I, where two NFA molecules form favorable hydrophobic contacts with each other and the extracellularly-oriented side of the Leu261 ring, thus blocking the ion permeation, whereas their carboxylate groups accept H-bonds from the Thr265 residues.

## Discussion

NFA is an anti-inflammatory drug clinically used for the relief of chronic and acute pain (Cremonesi and Cavalieri, 2015). In comparison with other nonsteroidal anti-inflammatory drugs or nonopioid analgesics, it is not associated with side effects, particularly, reactions in children (Sturkenboom et al., 2005). NFA is also widely used for inhibition of some types of Cl^−^-selective channels, blocking primary voltage-gated CLC-1 channels (Sturkenboom et al., 2005) and Ca^2+^-activated Cl^−^ channels (CaCCs) (White and Aylwin, 1990; Yang et al., 2008; Huanosta-Gutierrez et al., 2014).

It has been shown that a distinct branch of the CLC protein family, ClC-K kidney Cl^−^ channels, which are important for renal and inner ear trans-epithelial Cl^−^ transport (Zifarelli and Pusch, 2007), are modulated by NFA in a biphasic way: it activates ClC-K at low concentrations, but blocks the channels at high concentrations, above ~1 mM (Zifarelli et al., 2010). Guided by the crystal structure of a bacterial CLC homolog and site-directed mutations of ClC-K, authors suggested three amino acids as candidates for the potentiation effect of NFA. A subunit-specific NFA action has been demonstrated on the ligand-gated Cl^−^-permeable GABA_*A*_ receptors. Those, formed by α1/β2/γ2 subunits, the main receptor combination in the brain, were potentiated by application of NFA, while α6/β2/γ2 and α1/β2, α6/β2 receptors were inhibited by NFA (Sinkkonen et al., 2003). These observations suggest that NFA regulates the function of different Cl-selective channels via different pathways. However, molecular mechanisms of its action on Cys-loop receptors are elusive.

Here we used electrophysiological, mutational and molecular modeling analyses to investigate the effects of NFA on Cl^−^-selective GlyR channels of known subunit composition. We have demonstrated that NFA inhibits currents mediated by homomeric α1, α2, and α3 GlyRs with different efficacy: α1 receptors demonstrated the lowest affinity to NFA, while for α2 and α3 GlyRs the NFA affinity was more than ten times higher (Figures 2, 3). Inhibition of all three types of GlyRs by NFA was voltage dependent with higher affinity at positive potentials. This effect was especially pronounced for α2 GlyRs. The voltage dependence of NFA action allowed us to suggest that the site(s) of NFA interaction with GlyR are located in the channel pore. Moreover, the Woodhull analysis suggested that the NFA binding site in α1 GlyR is closer to the external part of the membrane, while for α2 GlyR it is significantly deeper in the pore.

The NFA ability to block α1 GlyRs at positive potentials significantly increased with the concentration of the glycine. In contrast, this effect in α2 GlyRs was not observed. We suggest that this difference is due to the different kinetics of channels' gating. Analysis of single-channel properties demonstrated that the mean open time of α2 GlyR channels exceeds that of α1 channels by almost 100-fold (Takahashi et al., 1992). Presumably, the different NFA potency in blocking the α1 GlyR currents induced by 30 and 100 μM of glycine is due to the different mean open times of the channel. Augmentation of the agonist concentration did not have an impact on the α2 GlyR block by NFA because in this GlyR subtype the mean open time even at low concentration of glycine was long enough for development of the maximum NFA effect.

The ion-conducting pore of GlyRs is lined by TM2 helices of five subunits. The TM2 segments of different alpha subunits differ only at the 2′ position: α1 receptors contain Gly, while α2 and 3 subunits have Ala (Figure 9A). The pore-blocking mechanism of NFA action suggests that the revealed difference in NFA sensitivity of different GlyRs is due, primarily, to different amino acids in the 2′ position.

In order to verify this, we have performed a point mutation in α1 subunit exchanging Gly254 for Ala. The G254A-α1 mutant was more sensitive to NFA than wild-type α1 GlyR and the NFA block in the mutant increased at positive potentials. However, this mutation did not convert completely the profile of α1 GlyR interaction with NFA to the one of α2 GlyR, suggesting that amino acids beyond the TM2 segments also influence the NFA action.

In our study, we have confirmed the importance of 2′ residue of TM2 segments in determining the action of pore-blocking molecules on GlyR. Several studies provided evidence of the implication of this amino acid in the interaction of GlyR with ion channel blockers (Pribilla et al., 1992; Rundstrom et al., 1994; Shan et al., 2001). For instance, cyanotryphenilborate (CTB) blocks α1 GlyRs more potently than α2 GlyRs, while mutation G254A in the α1 subunit makes the channel less sensitive to CTB (Rundstrom et al., 1994).

A prominent difference in the voltage dependence of α1 and α2 GlyRs block by NFA motivated us to implement the Woodhull analysis to determine the fractional electrical distance (δ) from the external side of the membrane at which NFA molecules bind to the α1, α2, and G254A-α1GlyRs. This analysis revealed that the δ value was largely different in different GlyR subtypes. Thus, the currents evoked by 100 μM of glycine were inhibited by NFA with higher potency likely due to the increase of the accessibility of deeper parts of the pore. A similar explanation is possible for the high NFA potency in α2 GlyRs. NFA molecules could penetrate to the pore by more than 60% of its length. The G254A-α1GlyR mutant, which had a higher NFA sensitivity than the α1 wild-type, also has a more extracellularly located NFA binding site.

Previous studies that considered voltage dependence of currents mediated by GlyRs formed a discrepant picture. Glycine-evoked currents demonstrated both an outwardly rectifying (Bormann et al., 1987; Schmieden et al., 1989; Morales et al., 1994; Downie et al., 1996; Fucile et al., 1999) and linear behaviors (Bormann et al., 1993; Wang et al., 2006) upon gradually changing V_hold_. This difference may be resulted from the use of different agonist concentrations and protocols of voltage change. Earlier study presented a clear explanation of these changes in I/V relations (Akaike et al., 1986).

A systematic study of I/V relations for different subunits of GlyRs was undertaken recently, but for desensitized channels, activated by high concentration of glycine (Raltschev et al., 2016). It was shown that currents mediated by α2 and α3 GlyRs in desensitized state are inwardly rectifying, while α1 GlyRs currents are linear. These results are partially overlapping with ours, but they cannot be fairly compared as Raltschev et al. (2016) studied desensitized receptors.

The Hill equation analysis demonstrated the different degree of cooperativity of NFA inhibitory action. It was higher for α1 GlyRs with n_H_close to 1.5 suggesting at least two molecules of NFA are necessary to block the pore, while for α2 GlyR n_H_ was close to 1. While there is no direct relation between Hill coefficients and stoichiometry of interaction, our observation that nH for α1 receptors is higher than for α2 receptors is in agreement with the proposed binding of two NFA molecules in the pore of alpha GlyR channels and supported by the model analysis.

Heteromeric α1β and α2β receptors were also inhibited by NFA. Incorporation of β subunit did not change significantly sensitivity of α1β GlyR to NFA, while at positive potentials NFA inhibited the α2β GlyR less potently than α2 receptors. The amino acid sequence of β subunit is very different from α subunits (Figure 9A). It was shown that Phe258 plays important role in determining the α1β sensitivity to PTX, and its substitution for threonine significantly increases the affinity of heteromeric receptors to PTX (Shan et al., 2001).

Our molecular modeling provided the possible structural rationale for the experimental data. The likely reason for the different strength, depth and stoichiometry of NFA binding in α1 and α2 GlyRs is favorable interactions of the hydrophobic ligand with the hydrophobic 2′ alanine ring in α2 GlyR and unfavorable interactions with the hydrated 2′ glycine ring in α1 GlyR. Being unable to bind at the 2′ glycine ring in α1 GlyR, NFA binds at the upper, wider levels of the pore that can accommodate two ligands. In a simplified way, NFA can be described as a molecule with two hydrophobic ends and a hydrophilic middle part (Figure 1). Such a molecule would favorably interact with the channel that has a hydrophilic ring with hydrophobic rings below and above it. The rings of Ala254, Thr258, and Leu261 residues in α2 GlyR do form such a pattern. α1 GlyR lacks such a pattern, and in the absence of the second hydrophobic ring, two NFA molecules enjoy hydrophobic contacts with each other. It should be noted that prediction of NFA binding sites in the big GlyR proteins would be hardly possible in a purely theoretical study. It is the experimental data on the mutational analysis of the pore-lining residue, the Hill coefficients of NFA action and the depth of the NFA binding sites revealed by the Woodhull analysis that provided strong experimental constraints to elaborate structural models of NFA action in α1 and α2 GlyRs.

In conclusion, several results of our study evidence in favor of the pore-blocking mode of NFA action on glycine receptors: (i) the voltage dependence of the block; (ii) increase of the α1 GlyR blocking efficacy with the increased glycine concentration; (iii) appearance of high frequency interburst flickering upon NFA application revealed by single channel recordings, and (iv) mutation G254A in the TM2 segment of α1 subunit increased the receptors sensitivity to NFA. Molecular modeling provided a possible structural rationale for our experimental observations and suggested several blocking sites of NFA with preferable sites between levels 2′ and 9′ in α2 GlyR and between levels 9′ and 13′ in α1 GlyR.

## Author contributions

GM and PB designed carried out and analyzed all patch clamp experiments, FP produced mutagenesis of glycine receptor and BZ performed molecular modeling.

### Conflict of interest statement

The authors declare that the research was conducted in the absence of any commercial or financial relationships that could be construed as a potential conflict of interest.

## Supplementary material

Supplementary Figure 1 **Representative traces of single channel currents from α2 GlyRs recorded in outside-out configuration of patch-clamp technique evoked by 5 μM of glycine (A)**, by mixture of 5 μM glycine+30 μM of NFA **(B)** and by glycine 5 μM+NFA 100 μM **(C)**; V_hold_ −30 mV.

Supplementary Figure 2 **IC_50_ curves for NFA at −80, −30, +30, and +80 mV obtained from current-voltage dependencies recorded during application of mixture of glycine with different NFA concentrations. (A)** Representative NFA IC_50_ curves for currents mediated by α1 GlyRs, induced by 30 μM of glycine; NFA IC_50_ at −80 mV = 272 μM, −30 mV = 256 μM, +30 mV = 218 μM, +80 mV = 178 μM. **(B)** Representative NFA IC_50_ curves for currents mediated by α1 GlyRs, induced by 100 μM of glycine; NFA IC_50_ at −80 mV = 209 μM, −30 mV = 171 μM, +30 mV = 107 μM, +80 mV = 94 μM. **(C)** Representative NFA IC_50_ curves for currents mediated by α1β GlyRs, induced by 30 μM of glycine; NFA IC_50_ at −80 mV = 191 μM, −30 mV = 200 μM, +30 mV = 152 μM, +80 mV = 144 μM. **(D)** Representative NFA IC_50_ curves for currents mediated by α2 GlyRs, induced by 30 μM of glycine; NFA IC_50_ at −80 mV = 155 μM, −30 mV = 75 μM, +30 mV = 17 μM, +80 mV = 12 μM. **(E)** Representative NFA IC_50_ curves for currents mediated by α2 GlyRs, induced by 100 μM of glycine; NFA IC_50_ at −80 mV = 100 μM, −30 mV = 39 μM, +30 mV = 12 μM, +80 mV = 7 μM. **(F)** Representative NFA IC_50_ curves for currents mediated by α2β GlyRs, induced by 30 μM of glycine; NFA IC_50_ at −80 mV = 84 μM, −30 mV = 47 μM, +30 mV = 24 μM, +80 mV = 22 μM. **(G)** Representative NFA IC_50_ curves for currents mediated by α3 GlyRs, induced by 100 μM of glycine; NFA IC_50_ at −80 mV = 117 μM, −30 mV = 78 μM, +30 mV = 23 μM, +80 mV = 10 μM. **(H)** Representative NFA IC_50_ curves for currents mediated by α1 G254A GlyRs, induced by 30 μM of glycine; NFA IC_50_ at −80 mV = 230 μM, −30 mV = 185 μM, +30 mV = 92 μM, +80 mV = 68 μM. **(I)** Representative NFA IC_50_ curves for currents mediated by α1 G254A GlyRs, induced by 100 μM of glycine; NFA IC_50_ at −80 mV = 260 μM, −30 mV = 190 μM, +30 mV = 106 μM, +80 mV = 57 μM.

## Abbreviations

CNS: central nervous system
cry-EM: cryoelectron microscopy
GlyR: Glycine Receptor
NFA: Niflumic acid
MC: Monte Carlo
MCM: Monte Carlo energy minimization
MP: membrane potential
V_hold_: holding potential.
