# Supplementary figures and images for: Voltage-Dependent Inhibition of Glycine Receptor Channels by Niflumic Acid
Source: Front Mol Neurosci. 2017 May 16;10:125. doi: 10.3389/fnmol.2017.00125 (PMC5432571; doi:10.3389/fnmol.2017.00125)

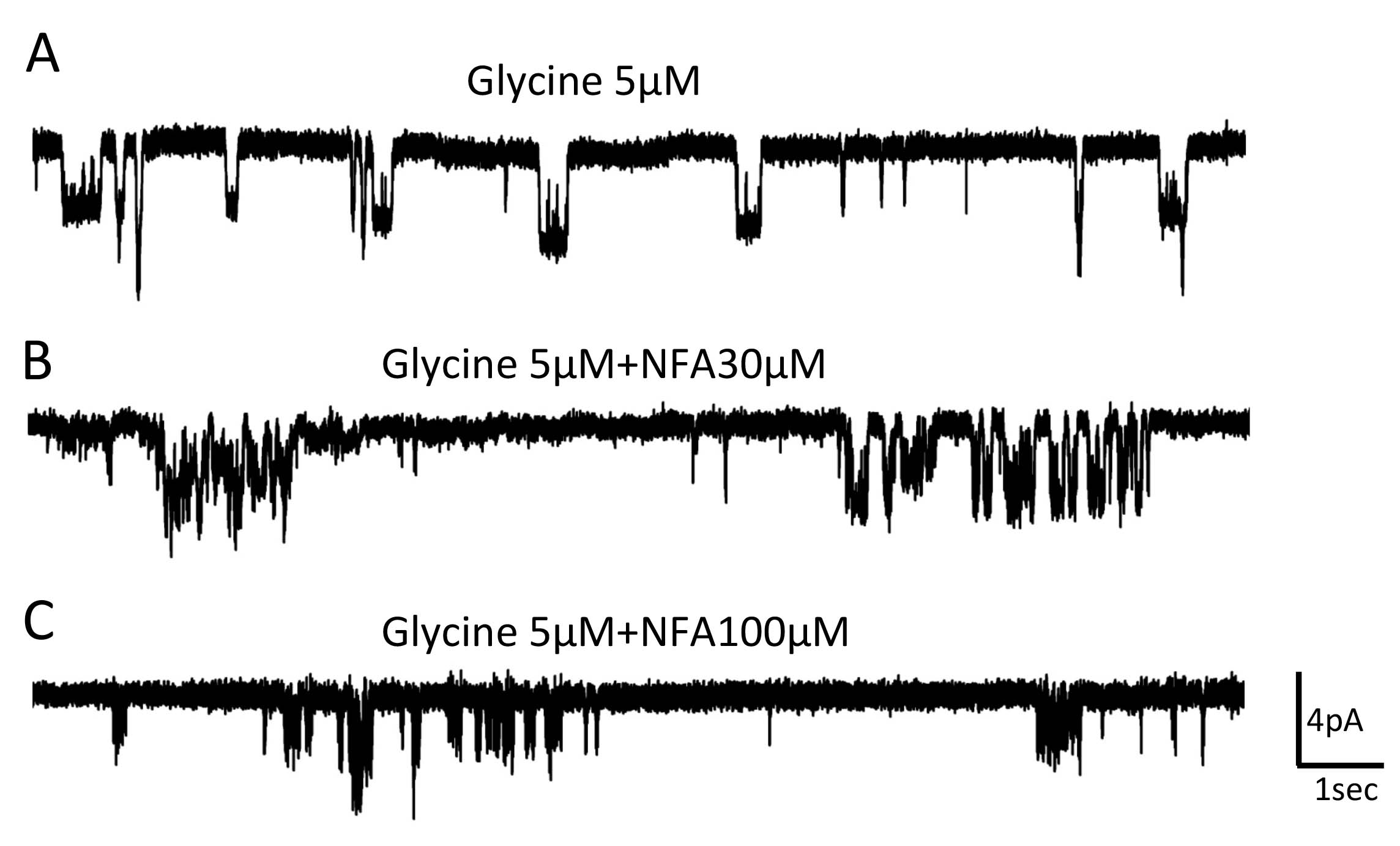

Supplement: Supplementary Figure 1 — Representative traces of single channel currents from α2 GlyRs recorded in outside-out configuration of patch-clamp technique evoked by 5 μM of glycine (A), by mixture of 5 μM glycine+30 μM of NFA (B) and by glycine 5 μM+NFA 100 μM (C); Vhold −30 mV. [file Image1.jpeg]

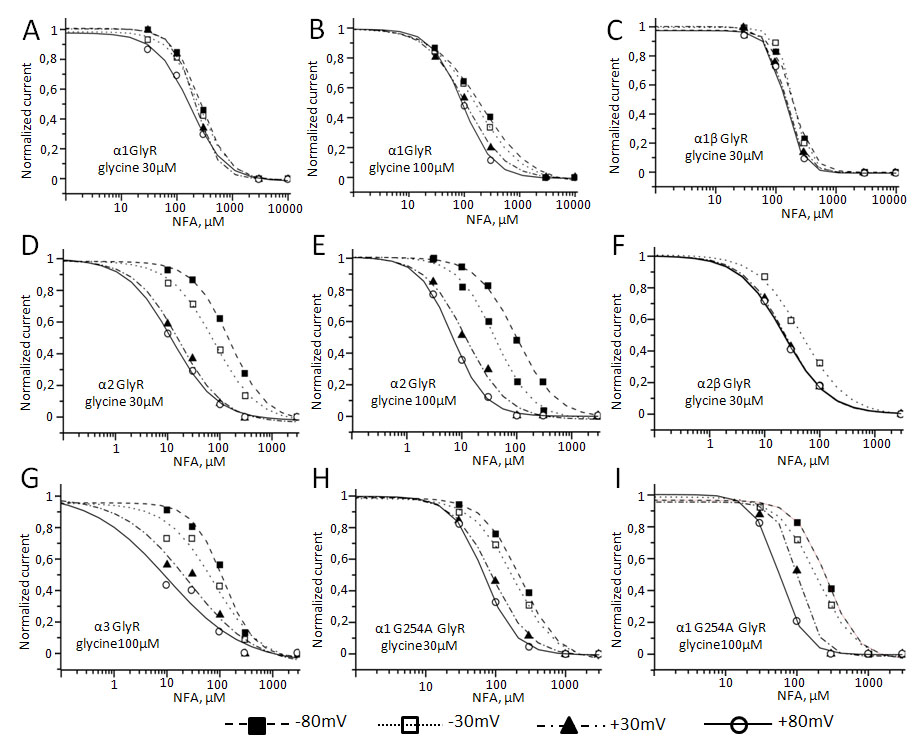

Supplement: Supplementary Figure 2 — IC50 curves for NFA at −80, −30, +30, and +80 mV obtained from current-voltage dependencies recorded during application of mixture of glycine with different NFA concentrations. (A) Representative NFA IC50 curves for currents mediated by α1 GlyRs, induced by 30 μM of glycine; NFA IC50 at −80 mV = 272 μM, −30 mV = 256 μM, +30 mV = 218 μM, +80 mV = 178 μM. (B) Representative NFA IC50 curves for currents mediated by α1 GlyRs, induced by 100 μM of glycine; NFA IC50 at −80 mV = 209 μM, −30 mV = 171 μM, +30 mV = 107 μM, +80 mV = 94 μM. (C) Representative NFA IC50 curves for currents mediated by α1β GlyRs, induced by 30 μM of glycine; NFA IC50 at −80 mV = 191 μM, −30 mV = 200 μM, +30 mV = 152 μM, +80 mV = 144 μM. (D) Representative NFA IC50 curves for currents mediated by α2 GlyRs, induced by 30 μM of glycine; NFA IC50 at −80 mV = 155 μM, −30 mV = 75 μM, +30 mV = 17 μM, +80 mV = 12 μM. (E) Representative NFA IC50 curves for currents mediated by α2 GlyRs, induced by 100 μM of glycine; NFA IC50 at −80 mV = 100 μM, −30 mV = 39 μM, +30 mV = 12 μM, +80 mV = 7 μM. (F) Representative NFA IC50 curves for currents mediated by α2β GlyRs, induced by 30 μM of glycine; NFA IC50 at −80 mV = 84 μM, −30 mV = 47 μM, +30 mV = 24 μM, +80 mV = 22 μM. (G) Representative NFA IC50 curves for currents mediated by α3 GlyRs, induced by 100 μM of glycine; NFA IC50 at −80 mV = 117 μM, −30 mV = 78 μM, +30 mV = 23 μM, +80 mV = 10 μM. (H) Representative NFA IC50 curves for currents mediated by α1 G254A GlyRs, induced by 30 μM of glycine; NFA IC50 at −80 mV = 230 μM, −30 mV = 185 μM, +30 mV = 92 μM, +80 mV = 68 μM. (I) Representative NFA IC50 curves for currents mediated by α1 G254A GlyRs, induced by 100 μM of glycine; NFA IC50 at −80 mV = 260 μM, −30 mV = 190 μM, +30 mV = 106 μM, +80 mV = 57 μM. [file Image2.jpeg]
